# Fatty Acid Inhibition Sensitizes Androgen-Dependent and -Independent Prostate Cancer to Radiotherapy via FASN/NF-κB Pathway

**DOI:** 10.1038/s41598-019-49486-2

**Published:** 2019-09-16

**Authors:** Hui-Yen Chuang, Yen-Po Lee, Wei-Chan Lin, Yi-Hsien Lin, Jeng-Jong Hwang

**Affiliations:** 1 Department of Biomedical Imaging and Radiological Sciences, National Yang-Ming University, Taipei, 112 Taiwan; 2 Department of Radiology, Cathay General Hospital, Taipei, Taiwan; 3 School of Medicine, Fu-Jen Catholic University, New Taipei City, Taiwan; 4 Division of Radiotherapy, Cheng Hsin General Hospital, Taipei, Taiwan; 5 School of Medicine, National Yang-Ming University, Taipei, Taiwan; 6 Department of Medical Imaging, Chung Shan Medical University Hospital, Taichung, Taiwan; 7 Department of Medical Imaging and Radiological Sciences, Chung Shan Medical University, Taichung, Taiwan

**Keywords:** Cancer metabolism, Radiotherapy

## Abstract

Elevated fatty acid synthase (FASN) has been reported in both androgen-dependent and -independent prostate cancers. Conventional treatment for prostate cancer is radiotherapy (RT); however, the following radiation-induced radioresistance often causes treatment failure. Upstream proteins of FASN such as Akt and NF-κB are found increased in the radioresistant prostate cancer cells. Nevertheless, whether inhibition of FASN could improve RT outcomes and reverse radiosensitivity of prostate cancer cells is still unknown. Here, we hypothesised that orlistat, a FASN inhibitor, could improve RT outcomes in prostate cancer. Orlistat treatment significantly reduced the S phase population in both androgen-dependent and -independent prostate cancer cells. Combination of orlistat and RT significantly decreased NF-κB activity and related downstream proteins in both prostate cancer cells. Combination effect of orlistat and RT was further investigated in both LNCaP and PC3 tumour-bearing mice. Combination treatment showed the best tumour inhibition compared to that of orlistat alone or RT alone. These results suggest that prostate cancer treated by conventional RT could be improved by orlistat *via* inhibition of FASN.

## Introduction

Prostate cancer (PCa) is the most common malignant neoplasm among men. Early stage PCa is slow-growing cancer with a better prognosis compared with other cancers. Both the prostate-specific antigen (PSA) assay and digital rectal examination (DRE) are used routinely for PCa detection^1^. Radiotherapy (RT) is one of the primary PCa treatments and shows comparable cure rate to radical prostatectomy when treating localised low-grade PCa. RT alone or combined with androgen deprivation therapy (ADT) are also used to control and relieve symptoms in patients with advanced and metastatic PCa. Many patients respond to ADT at the beginning because androgen is essential for early PCa. However, ADT becomes ineffective when PCa develop into androgen refractory and castration-resistant (CR) status, and the median survival time significantly decreased^2^. Thus, better treatments for PCa is urgently needed.

Like many cancers, PCa shows upregulated lipogenesis^3^, which strongly affects androgen synthesis. Downregulations of lipogenesis-related genes and enzymes, including fatty acid synthase (FASN)^4^, 3-hydroxy-3-methylglutaryl-CoA synthase (HMG-CoA synthase)^5^, and sterol-regulatory-element-binding protein-1c/2 (SREBP-1c/2)^6^ have been shown to inhibit cancer growths significantly. Additionally, the lipid is critical for the stability of lipid rafts, where different receptors involving in various signalling cascades are located, especially, in cancer cells^7^. Lipid raft disruptions^8^ and cell membranes modifications by adding excess polyunsaturated fatty acids have been shown to inhibit the tumour growth^9,10^.

Differ from other cancer, PCa mainly generates energy through lipid β-oxidation rather than aerobic glycolysis. Increased lipid levels further accelerate cell proliferation and angiogenesis, resulting in the more hypoxic and acidic tumour microenvironment, and lead to poor prognosis^11,12^. Both hypoxia and angiogenesis are critical for the development of radioresistance. Another factor strongly related to radioresistance is p53, a tumour suppressor, which determines the cell fates after DNA damages induced by ionising radiation (IR) or chemotherapy. The status of p53 determines whether cells arrest at the G1 phase or enter apoptosis process after FASN inhibition^13^. However, how FASN affects the radiosensitivity in PCa cells with different p53 statuses has not been well studied. Transduction of wide-type p53 sensitised p53^wild type^ LNCaP and p53^null^ PC3 to radiation, suggesting functional p53 may enhance cell radiosensitivity^14^.

On the contrary, Scott *et al*. demonstrated that p53 restoration decreased radiosensitivity in PC3 cells^15^. Other than p53 status, cell radiosensitivity is also controlled by IR-induced Akt^16^, ERK^17^, and NF-κB^18^. Improved RT outcomes caused by NF-κB inhibition have been seen in oral and colorectal cancer, and Akt inhibition-mediated radiosensitisation has also been observed in glioma^19^ and prostate cancer^20^. Moreover, a positive feedback loop between PI3K/Akt and FASN has been proposed^21,22^.

FASN inhibition by C75 or *si*FASN significantly suppressed PCa growth *in vivo*^23^. Moreover, orlistat, a FASN inhibitor slowed tumour growth and altered cancer metabolism as observed by the ^11^C-acetate/microPET imaging in xenograft mice^24^. Cancer metabolism strongly affects RT outcomes by shaping the tumour microenvironment. For instance, tumour hypoxia and acidity resulted in HIF-1α and VEGF expressions, radioresistance, and treatment failure^25,26^.

In this study, we found that orlistat decreases NF-κB activity and its downstream effector expressions in androgen-dependent LNCaP and androgen-independent PC3 cells. Our results also suggest that FASN inhibition improves RT outcomes in PCa through NF-κB suppression. Moreover, the combination of orlistat and RT shows the most tumour inhibition in both LNCaP and PC3 xenograft mice. Therefore, we propose that FASN inhibition enhances RT outcomes and the possible mechanisms and the combination of FASN inhibition and RT could be a novel strategy for PCa treatment.

## Results

### Orlistat reduces cell proliferation via arresting cell cycle progression and the radiation survival curves of LNCaP and PC3 cells

**Figure 1:**
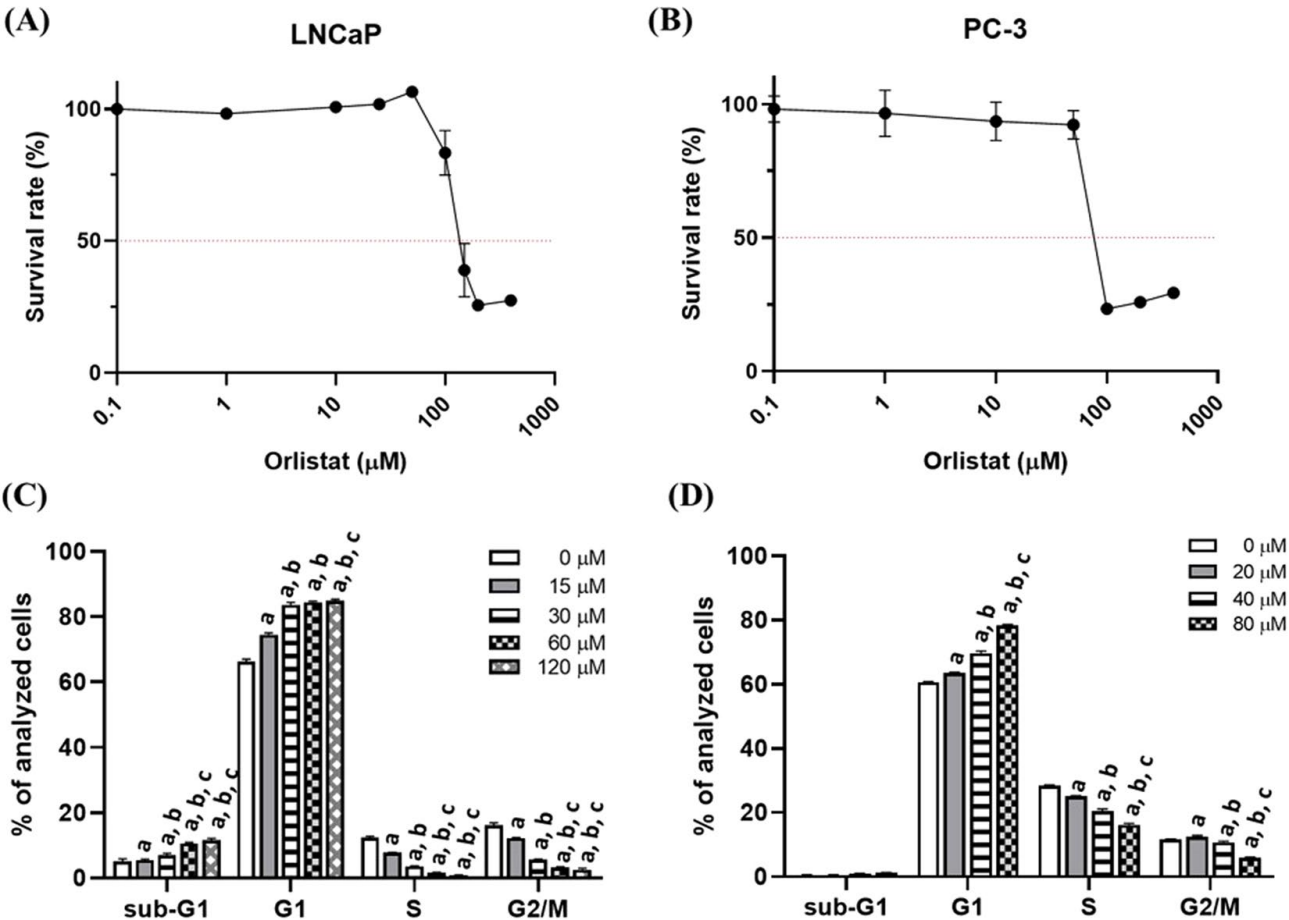
Orlistat suppressed proliferation and caused cell cycle changes in LNCaP and PC3 cells. Orlistat resulted in significant G1 phase accumulations and S and G2/M phase reductions in both (**C**) LNCaP and (**D**) PC3 cells in a dose-dependent manner. Moreover, orlistat also caused increases in the sub-G1 population in LNCaP cells but not in PC3 cells. (*a*, p < 0.05 compared with 0 μM; *b*, p < 0.05 compared with 15 μM; *c*, p < 0.05 compared with 30 μM; *d*, p < 0.05 compared with 60 μM; *e*, p < 0.05 compared with 20 μM; *f*, p < 0.05 compared with 40 μM).

**Figure 2:**
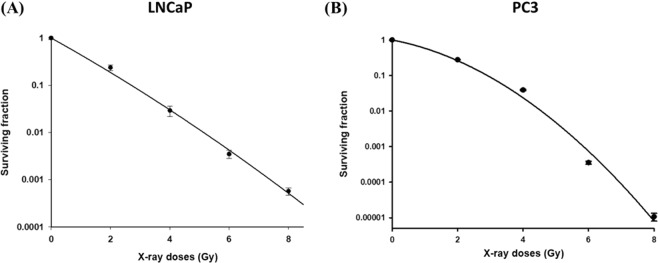
Radiation survival curves of LNCaP and PC3 cells. Radiation surviving curves of (**A**) LNCaP and (**B**) PC3 cells were built based on the colony formation results, and the D_1_ of both cell lines are calculated. The D_1_ values of LNCaP and PC3 cells were 1.1 Gy and 2 Gy, respectively. The D_1_ of both cell lines were used in the following combination treatments.

Cytotoxicities of orlistat in LNCaP and PC3 cells were determined by AlamarBlue assay after 48 hours of orlistat treatment. The IC_50_ of orlistat in LNCaP and PC3 cells were 120 µM and 80 µM, respectively (Fig. 1A,B). Figure 1C,D exhibit the changes in the cell cycle caused by orlistat in both cell lines. Significant accumulation in G1 phase and reduction in S phase were found in both cell lines; however, the insignificant sub-G1 population was found in PC3 cells. Radiation survival curves and the D_1_ of both cell lines were obtained from the colony formation results. The D_1_s, *i*.*e*., the dose required to reduce the fraction of surviving cells to 0.37, were 1.1 Gy and 2 Gy for LNCaP cells and PC3 cells, respectively (Fig. 2A,B). The D_1_ obtained from colony formation assay was used for the following experiments.

### Orlistat decreases NF-κB activities and changes in protein expressions

**Figure 3:**
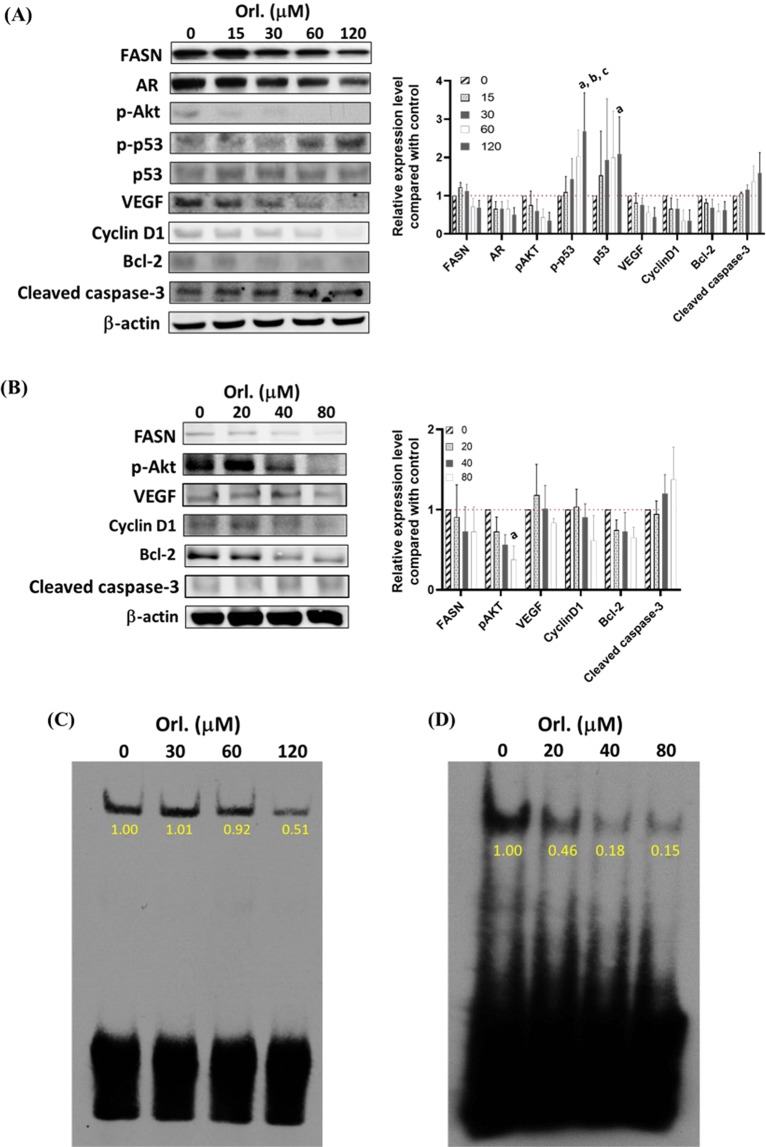
Orlistat alters the protein expression profiles and the NF-κB activity in LNCaP and PC3 cells. The protein expressions were evaluated by Western blotting. (**A**,**B**) Orlistat reduced the expressions of FASN, pAkt, VEGF, cyclin D1, and Bcl-2 in both cell lines. Additionally, slightly increased cleaved caspase-3 was also detected. Decreased androgen receptor (AR) and elevated p-p53/p53 levels were found in LNCaP cells after orlistat treatment. (**C**,**D**) NF-κB activity was determined by the EMSA assay, and orlistat inhibited the NF-κB activity in both cell lines in a dose-dependent manner. PC3 cells showed a more profound reduction in NF-κB activity at the dose of IC_50_ than LNCaP cells. (a, p < 0.05 compared with 0 μM; b, p < 0.05 compared with 15 μM; c, p < 0.05 compared with 30 μM).

The protein expression profiles were altered by orlistat in both cell lines as shown by Western blotting (Fig. 3A,B). Proteins including fatty acid synthase (FASN), pAkt, VEGF and cyclin D1 were inhibited by orlistat in a dose-dependent manner in both cell lines. The protein level of the androgen receptor (AR) was decreased, and p-p53 was increased in LNCaP cell line. Apoptosis-related cleaved caspase-3 was increased, while anti-apoptosis Bcl-2 were decreased in a dose-dependent manner in both cell lines. Since VEGF, cyclin D1 and Bcl-2 were regulated by NF-κB, EMSA was performed to determine the NF-κB activities in both cell lines. The results showed that NF-κB activities were decreased in a dose-dependent manner in both cell lines; however, the inhibition was more profound in PC3 cells (Fig. 3C,D).

Effects of combination treatment on NF-κB activity and protein expressions in LNCaP and PC3 cells.

**Figure 4:**
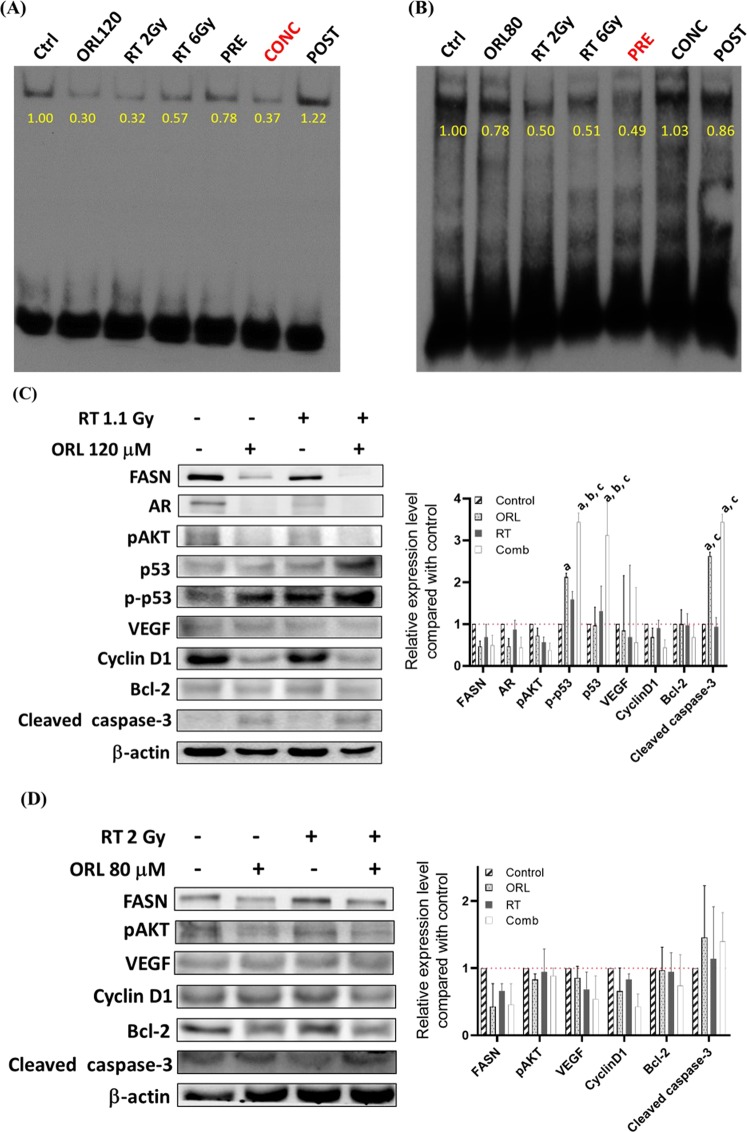
NF-κB activity change was determined by EMSA assay, and the changes in protein expression profiles were detected by Western blotting. Lower NF-κB activities were found in orlistat and RT-treated (**A**) LNCaP and (**B**) PC3 cells. The lowest NF-κB activity among all the combination groups was detected in the concurrent group and pretreated group in LNCaP and PC3, respectively. According to the EMSA findings, LNCaP and PC3 cells were received concurrent and pretreatment for the subsequent Western blotting. (**C**) RT slightly reduced FASN, AR, and cyclin D1 expressions in LNCaP cells and these reductions were further enhanced by the combination treatment. Also, combination treatment increases p53 and p-p53. (**D**) Similar effects on protein expression changes were also observed in PC3 cells except for AR, p53, and p-p53, which are not expressed by PC3 cells.

Here we used EMSA as the endpoint to determine the optimal strategy for combination treatment. Figure 4A,B show the NF-κB activity was decreased in LNCaP cells but increased in PC3 cells 48 hours after X-ray irradiation. The lowest NF-κB activities in LNCaP and PC3 cells were found in the concurrent and pretreatment, respectively. Therefore, these two treatment schedules were used for the following experiments. The radiation and orlistat decreased the expressions of FASN, pAkt, VEGF, and cyclinD1 in LNCaP and PC3 cells. (Fig. 4C). The combination treatment further enhanced the changes in protein expressions induced by orlistat or RT alone in both cell lines.

### Orlistat combined with RT enhances the therapeutic efficacy in LNCaP and PC3 tumour-bearing mouse models

**Figure 5:**
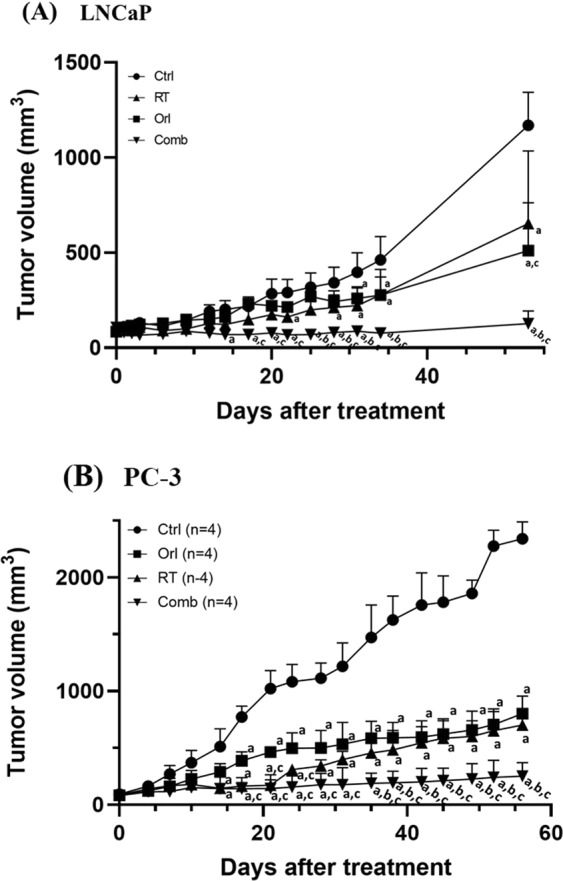
Orlistat synergistically improves RT outcomes in both LNCaP and PC3 tumor-bearing mice. The combination treatment significantly suppressed (**A**) LNCaP and (**B**) PC3 tumor growth. Significant differences in tumor sizes were found between COMB, ORL, and RT groups as indicated in the figure. (a, p < 0.05 compared with Control; b, p < 0.05 compared with orlistat; c, p < 0.05 compared with RT).

Mice with subcutaneous LNCaP or PC3 tumours were randomly divided into four groups (n = 4–5 mice per group) and received different treatments as described in the Materials and methods. Figure 5A shows that the best tumour therapeutic efficacy in LNCaP tumour-bearing mice was found in the combination (COMB) group, and the tumours were almost totally inhibited with a significantly smaller mean size than other groups from Day 21 to Day 32. Both orlistat and RT alone exhibited similar inhibition effects on LNCaP tumours. Figure 5B exhibits the tumour growths in PC3 tumour-bearing mice. Both orlistat and RT showed great tumour suppressions as compared with the CTRL group, and both groups showed significant differences compared with the CTRL group from Day 14 after treatment. RT irradiation had better tumour inhibition than orlistat and kept the tumour sizes close to that of the COMB group in the first twenty days after treatment. However, the tumours received RT began to grow rapidly after Day 45 and had no difference when compared with the ORL group at the following time points.

## Discussion

Androgen functions as the fuel for the growth of prostate cancer (PCa), and androgen deprivation therapy (ADT) is applied to patients to slow the tumour growth *via* blockade of the androgen-related signalling pathway. Although most patients respond to ADT at the beginning, some PCa turns into castration-resistant (CR) form and results in extremely inferior prognosis. Radiotherapy (RT) is delivered to PCa patients with early or late stages for various purposes. Moreover, it has been shown that the androgen receptor (AR) signalling pathway affects the radiosensitivity in bladder cancer^27^ and prostate cancer^28^. The clinical results also indicated that ADT could enhance the effects of RT on PCa. These findings suggest that androgen may play a role in modulating the radiosensitivity of PCa to ionising radiation.

It is known that androgen is primarily made from dehydroepiandrosterone (DHEA), which is derived from cholesterol *via* four-steps synthesis. Thus, disrupting lipogenesis seems to be a potential way to inhibit the PCa progression since lipogenesis is highly upregulated in PCa. FASN is one of the critical enzymes in lipogenesis. It has been shown that FASN inhibition could arrest the PCa growth both *in vitro* and *in vivo*^23,29,30^. Recently, Zadra *et al*. demonstrated that knockdown of FASN reduced the AR activity in androgen-dependent and CR form of PCa. The authors further proved that FASN inhibition suppressed cell proliferation through reprogramming cancer metabolism and inducing ER stress^31^. Here we used orlistat as a FASN inhibitor and combined with ionising radiation to investigate the combinatorial effects. Figure 1 exhibits that orlistat suppressed cell growth in both androgen-dependent LNCaP and androgen-independent PC3 cells with IC_50_ = 120 μM and 80 μM, respectively. Significant accumulation of G1 phase and reduction of S phase were found in both cell lines in a dose-dependent manner (Fig. 1C,D). S phase is known as the most radioresistant phase of the cell cycle^32^, and reduced S phase population may enhance the radiosensitivity of cells. G1 phase blockage caused by FASN inhibition has been reported in several cancer types^24,31,33,34^. The bidirectional regulation of AR and cell cycle has been reported by several groups. Yuan *et al*. found that *sh*RNA-mediated AR downregulation resulted in G1 arrest through increasing p27 and decreasing p-pRb expressions in androgen-independent CWR22 cells^35^. AR could push the cell cycle forward in LNCaP cells by forming the pre-replication complex, which further cooperates with cyclin A and be used for DNA replication machinery^36^. Moreover, AR not only triggers G1-S transition but also promotes the expression of dihydroceramide desaturase 1 (DEGS1), which could be used as a progression biomarker^37^.

Here the radiation survival curves were built using the results obtained from the colony formation assay on both LNCaP and PC3 cells. The D_1_s obtained from the radiation survival curves are 1.1 and 2 Gy for LNCaP and PC3 cells, respectively (Fig. 2A,B). These two doses were used for the following experiments to evaluate the combination effects. It is worth noting that LNCaP showed a linear radiation survival curve which was also found in other studies^38,39^. The linear survival curve implies that LNCaP cells might lack double-strand break repair (DSB repair) capability. Collis *et al*. proved that LNCaP could not correctly repair the DSB with a rapid dual fluorescent assay^40^. Besides, results obtained by Turney *et al*. also indicated that LNCaP might lack DSB repair by knocking down type 1 insulin-like growth factor receptor (IGF-1R), which regulates DSB repair. The authors found that knockdown of IGF-1R sensitised PC3 and DU145 but not LNCaP cells to ionizing radiation^41^.

We further calculated the mean lethal doses D_0_ of LNCaP and PC3 cells, which are 1.2 Gy and 0.7 Gy, respectively. D_0_ is known as the mean lethal dose and represents the radiosensitivity of cells. The finding is similar to the results recently reported by Ide *et al*.^27^, suggesting that the androgen receptor (AR) signaling reduces radiosensitivity of cells. RT combined with ADT achieves the better outcomes than RT alone in PCa patients, and the underlying mechanisms have been discovered. Tarish *et al*. further demonstrated that castration or ADT improves RT responses through impairing DNA DSB repair^42^. Spratt and colleagues found the AR-overexpressed LNCaP-AR cells are more resistant to radiation than the parental LNCaP cells. Higher AR expressions are correlated to more DNA repairs after irradiation^43^.

Moreover, their results also showed that prostate-specific antigen (PSA), TMPRSS2, and KLK2 are upregulated after irradiation. Elevated PSA is usually found in those patients post-ADT due to the failure of the primary therapy^43,44^. Besides, we knocked-down the FASN using *sh*FASN and performed colony formation to prove that FASN could regulate the radiosensitivity in PCa. The *sh*FASN-transduced LNCaP cells showed significantly lower survival rate than parental LNCaP cells when treated with the same X-ray doses (>6 Gy) (Fig. S2), suggesting the correlation between FASN expression and radiosensitivity.

The FASN expression levels between LNCaP and PC3 cells were compared using Western blotting (Fig. S3). LNCaP cells expressed more FASN protein than PC3. It has been found that the FASN expression level does not correlate to the sensitivity to FASN inhibitor^33^, which also shown in Fig. 1A,B. Orlistat slightly increased cleaved caspase-3, decreased Bcl-2, and cyclin D1 expressions in both cell lines (Fig. 3A,B). Reduction of pAkt was also found after orlistat treatment. The PI3k/Akt/FASN pathway in PCa was first demonstrated by Van de Sande and colleagues. In their study, both the activity and expression of FASN were suppressed in LNCaP cells by treating with LY294002, a PI3k inhibitor.

Furthermore, the reduced FASN activity could be rescued by transfecting the construct encoding constitutively active Akt^37,45^. The high-fat diet induces the Akt-FASN activation and promotes tumour progression. A negative correlation of serum FASN reduction and prognosis was observed after ADT in PCa patients^46^. Our results showed that the expression of AR was decreased after FASN inhibition, suggesting that a two-way modulation between AR and FASN (Fig. 3A). The EMSA assay demonstrated NF-κB activity inhibited by the orlistat treatment in a dose-dependent manner in both cell lines (Fig. 3C,D). Increased NF-κB activity results in radioresistant and chemoresistant in various cancer types^47^, and its inhibition could diminish the resistances^48–50^. NF-κB is known as a signalling hub and regulates multiple effector proteins, including VEGF, cyclin D1, and Bcl-2. The expressions of these effector proteins were decreased as shown in this study. Here we have shown that the NF-κB activity can be suppressed by FASN inhibitor to enhance the tumour control when combined with radiotherapy. In contrast to our finding, Menendez *et al*. found that FASN inhibition lowered p53 expression and activated MEK, ERK, and nucleus translocation of NF-κB in breast cancer^51^.

The NF-κB activity strongly correlates to the radioresistance, and its activity was examined by EMSA to determine the optimal strategy for the combination treatment. Since both concurrent and pretreatment showed the lowest NF-κB activity, these two strategies were applied to LNCaP and PC3 cells, respectively (Fig. 4A,B). Combination treatment further enhanced the protein changes after treatments in both cell lines (Fig. 4C,D). Orlistat increased more cleaved caspase-3 expression in LNCaP than PC3, which echoed the results obtained from flow cytometry showing more sub-G1 population in LNCaP cells (Fig. 1C,D). Kao *et al*. reported that the FASN level is correlated to radioresistance and could be used as a prognostic marker in nasopharyngeal carcinoma^52^. The correlation of radioresistance to FASN activity was also observed in pancreatic cancer^53^. Another FASN inhibitor C75 also was shown to sensitise PCa cells to RT^54^ in cell culture. Differing from the orlistat that only targets the thioesterase domain of FASN, C75 mainly acts on the β-ketoacyl synthase domain as other FASN inhibitors. It has also been reported that C75 could target the enoyl reductase and thioesterase domains. C75 is the analogue of natural compound cerulenin^55^. Although C75 shows potent tumor inhibition, it affects the fatty acid synthesis in normal liver and some side effects on normal tissues^56^. In addition, C75 reduces the food intakes in rodents and results in the body weight loss because it also targets carnitine palmitoyltransferase I (CPT1)^57^.

We applied the combination of orlistat and radiation to both LNCaP and PC3 tumour-bearing mouse models and found that the most significant tumour control as compared with that of RT alone or orlistat alone in both mouse models. The combination treatment inhibited LNCaP tumour growth even after orlistat was removed on day 31 post initiation of the treatment (Fig. 5A). Significant differences were found in both RT and COMB groups as compared with the CTRL group in the first 10-day after treatment in PC3 tumour-bearing mice. Better tumour inhibition by RT might be owing to higher radiation dosage given to PC3 tumour-bearing mice compared with that given to LNCaP tumour-bearing mice. Interestingly, PC3 tumours seemed to be more sensitive to orlistat similar to those found in the cell culture.

Moreover, tumours in the COMB group began significantly smaller compared with the ORL group from day 20 after treatment and were significantly smaller compared with the other groups after day 40 (Fig. 5B). These results imply that orlistat-mediated tumour inhibition is not only through FASN inhibition but other signalling regulators such as AR and NF-κB as shown in this study. We further calculated the growth inhibition rates, enhance rates, and combination indices (CIs) to evaluate the efficacy of combination treatment^58^ compared with RT or orlistat alone in both LNCaP and PC3 cells as shown in Tables 1 and 2. The CIs for both tumour-bearing mouse models are <1, indicating that the combination treatment is synergistic compared with RT or orlistat treatment alone.

**Table 1:** Tumour growth inhibition of LNCaP tumour-bearing mice after received different treatments.

Group	Numbers of mice	Mean tumour growth time (day)^a^	Mean tumour growth delay time (day)^b^	Mean growth inhibition rate^c^	Enhance rate^d^	Mean growth inhibition rate^e^	Expected growth inhibition rate (%)^f^	Combination index^g^
Control	4	13.7	N/A	N/A	N/A	—	—	—
ORL	4	15.6	1.9	1.1	2.4	31	—	—
RT	4	27.8	14.1	2	1.3	32	—	—
Comb	5	51.4	37.8	3.8	1	88	53	0.3

^a^Mean tumour growth time: the time at which the tumour volume reaches to 200 mm^3^.

^b^Mean tumour growth delay time: the tumour growth time of the treated group minus that of the

Control group.

^c^Mean growth inhibition rate: the growth rate of treated group/ growth rate of Control.

^d^Enhance rate: growth inhibition rate of Comb group/ growth inhibition rate of the treated group.

^e^Mean growth inhibition rate: the maximum mean tumour volume of treated group/ the maximum mean tumour volume of Control.

^f^Expected growth inhibition rate: inhibition rate of ORL & RT plus together, and then minus inhibition rate of ORL & RT multiply.

^g^Combination index:(1-Mean growth inhibition rate of Comb)/ (1-Expected growth inhibition rate).

**Table 2:** Tumour growth Inhibition of PC3 tumour-bearing mice after received different treatments.

Group	Numbers of mice	Mean tumour growth time (day)^a^	Mean tumour growth delay time (day)^b^	Mean growth inhibition rate^c^	Enhance rate^d^	Mean growth inhibition rate^e^	Expected growth inhibition rate (%)^f^	Combination index^g^
Control	4	8.6	N/A	N/A	N/A	—	—	—
ORL	4	18.4	9. 8	2.1	2.4	33	—	—
RT	4	34.3	25.7	4	1.3	43	—	—
Comb	4	43.6	35	5.1	1	81	62	0.5

^a^Mean tumour growth time: the time at which the tumour volume reaches to 200 mm^3^.

^b^Mean tumour growth delay time: the tumour growth time of the treated group minus that of the

Control group.

^c^Mean growth inhibition rate: the growth rate of treated group/ growth rate of Control.

^d^Enhance rate: growth inhibition rate of Comb group/ growth inhibition rate of the treated group.

^e^Mean growth inhibition rate: the maximum mean tumour volume of treated group/ the maximum mean tumour volume of Control.

^f^Expected growth inhibition rate: inhibition rate of ORL & RT plus together, and then minus inhibition rate of ORL & RT multiply.

^g^Combination index:(1-Mean growth inhibition rate of Comb)/ (1-Expected growth inhibition rate).

Orlistat is an FDA-approved anti-obesity drug due to its lipase inhibitor capability (IC_50_ = 122 ng/ml). The IC_50s_ of orlistat as a FASN inhibitor in LNCaP and PC3 cells are significantly higher than the IC_50_ for orlistat as a lipase inhibitor. Off-target effects due to the differences in IC_50_ may be concerned. Hence, body weights were also monitored to assess the general toxicities caused by treatments, and no significant differences or reductions in body weights were found in all groups. Insignificant body weight change may be due to the nutrient compositions in mouse chow, which generally contains 4–4.5% of fat. Orlistat reduces body weights of subjects by blocking fat uptakes. In other words, orlistat would not cause body weight reduction if the fatty acids were relatively low in the food.

Additionally, orlistat has poor bioavailability and short *in vivo* half-life. According to the information provided by the FDA drug database, orlistat has poor bioavailability and short *in vivo* half-life (1–2 hr), and no adverse side-effects were observed in healthy subjects receiving doses above 120 mg three times a day. Recently, orlistat has been shown as a multitargeted agent for cancer therapy. The potential targets include ribosomal proteins 7a, 9, and 14 (RPL 7a, 9, and 14), β-tubulin, GAPDH, and Annexin A2^59^. These proteins modulate tumour progression by modulating genomic and chromosomal stability, glycolysis, and membrane trafficking^60^. Even though more experiments have to be carried out to conclude that radiosensitization-mediated by orlistat may also be related to these proteins.

In conclusion, this is the first study, to our knowledge, demonstrates that FASN inhibition could sensitise prostate cancer cells to ionising radiation both *in vitro* and *in vivo*. Our results suggest that FASN inhibition improves the outcomes of radiotherapy by redistributing the cell cycle and downregulating the androgen receptor signalling in LNCaP cells. Furthermore, suppression of the NF-κB activity *via* inhibition of FASN in both LNCaP and PC3 cells may provide a novel strategy for radiotherapy of the prostate cancer.

## Materials and Methods

### Cell culture

Androgen-dependent and -independent human prostate cancer cell lines, LNCaP and PC3, were cultured in RPMI-1640 (Hyclone) and F-12K (Corning) medium containing 10% FBS (Hyclone) and 1% penicillin/streptomycin (Corning), respectively. Both cells were maintained at 37 humidified CO_2_ incubator.

### Drug preparation

For *in vitro* experiments, the 40 mM orlistat stock solution was obtained by dissolving a Xenical capsule in 6 ml absolute ethanol (Merck) and stored at −20 °C. For *in vivo* treatment, orlistat was dissolved in 33% absolute ethanol and 66% PEG400 (Sigma). The mice were treated with 240 mg/kg body weight/day^24^.

### Cytotoxicity assay

1.5 × 10^4^/well LNCaP and 1 × 10^4^/well PC3 cells were seeded into 96-well plates and treated with various doses of orlistat for 48 hours. The cytotoxicity of orlistat in both cell lines was assessed by AlamarBlue assay (Pierce). Briefly, 20 µl (*i*.*e*., 1/10 volume of the culture medium) of AlamarBlue reagent was added into each well. The plate was assayed at 570 and 600 nm with an ELISA reader (TECAN Sunrise) after 4-hour incubation at 37 °C. The cell viability was calculated by the equation provided by the manufacturer.

### X-ray irradiation

7.5 × 10^5^ LNCaP and 5 × 10^5^ PC3 cells were seeded into 6-cm dishes one day before irradiation with cabinet X-ray irradiator (RS-2000, Rad Source Technologies Inc.). Cells exposed to 0–8 Gy IR were kept on ice before seeding. Different numbers of cells were seeded into 6-cm dishes according to the irradiated doses. Colonies were fixed, stained, and counted under dissecting microscope after a 14-day incubation. Only those colonies with more than 50 cells were counted to establish the radiation survival curves, and the D_1_ of each cell line was obtained and used for the following experiments.

### Cell cycle analysis

7.5 × 10^5^ LNCaP and 5 × 10^5^ PC3 cells were seeded into 6-cm dishes before 48 hr of orlistat treatment. Cells and medium were collected, centrifuged, and washed with PBS. Cells were fixed using cold 70% ethanol/PBS and incubated at −20 °C for at least 24 hours before propidium iodide staining (10 µg/ml in PBS). The results were collected by the FACSCalibur (BD Bioscience) and analysed with the FlowJo software (Tree Star).

### Western blotting

2 × 10^6^ LNCaP and 1.5 × 10^6^ PC3 cells were seeded into 10-cm dishes one day before orlistat treatment. Cells were collected and lysed with NP-40 buffer containing Halt Protease inhibitor cocktail (Thermo Fisher Scientific). Based on the molecular weights of the proteins of interest, 30–40 µg lysates were separated by 8–15% SDS-PAGE and transferred to PVDF membranes. Membranes were blocked with 5% nonfat milk at room temperature for 1 hour before incubated with primary antibodies overnight at 4 °C. Membranes were washed with TBS-T and incubated with HRP-conjugated secondary antibodies (Jackson ImmunoResearch Laboratories) at room temperature for 1 hour. ECL chemiluminescent detection system (Millipore) was used for the final detection, and signals were acquired by ImageQuant LAS4000 (GE Healthcare Life Science). β-actin was served as a loading control. Quantitative analysis was performed by ImageJ (National Institutes of Health).

### Electrophoretic mobility shift assay (EMSA)

Nuclear proteins were isolated from LNCaP and PC3 cells using Nuclear Extraction kit (Chemicon International) according to the manufacturer’s protocol. The NF-κB/DNA binding activity was evaluated using the LightShift Chemiluminescent EMSA kit (Pierce). The following DNA sequences were synthesised for EMSA analysis: *AGTTGAGGGGACTTTCCCAGGC* (sense) and *GCCTGGGAAAGTCCCCTCAACT* (antisense)^17^ and were further labeled with biotin. Briefly, nuclear proteins were incubated with biotin-labeled DNA probes for 20 min then electrophoresed on 5% polyacrylamide gel. Then transferred to a nylon membrane and cross-linked by UV light. Signals were detected by ECL provided by the EMSA kit after streptavidin-horseradish peroxidase incubation and analysed by ImageJ for quantitative results.

### Animal model and treatment evaluations

All the animal experiments were approved by the IACUC of National Yang-Ming University with the protocol number: 1031256. All methods were performed in accordance with the relevant guidelines and regulations. 6 × 10^6^ LNCaP or 2 × 10^6^ PC3 cells mixed with standard Matrigel (Corning) were inoculated into the right thighs of 6 to 8-week-old male nude mice. Mice were randomly separated into the following groups (n = 4–5) when the mean tumour volume reached 100 mm^3^: control (CTRL), 240 mg/kg/day orlistat (ORL), X-ray irradiation (RT) and combination (COMB) groups, as shown in Fig. S4. 240 mg/kg/day orlistat and the solvent (*i*.*e*., 33% ethanol/ 66% PEG400) were given *via* intraperitoneal injection daily to ORL and CTRL groups, respectively. A single dose of 3 Gy or 6 Gy X-ray irradiation was given to LNCaP and PC3 tumour mice, respectively. The COMB group received the first dose of orlistat 2 hours before X-ray irradiation and other daily doses of orlistat from the next day. Tumour volumes and body weight were monitored to evaluate the treatment responses and general toxicity. Tumour sizes were calculated using the formula:*length* × *width*^2^ × 0.523^61^. All the animal studies were performed twice.

### Statistics

All the results were presented as mean ± standard deviation (S.D.). The statistics of cell cycle, Western blotting and animal study results were performed using two-way ANOVA, followed by Tukey’s post hoc test. The results were plotted using Prism 8 (GraphPad).

## Supplementary information

Supplemental information
