# Supplementary material for: Fatty Acid Inhibition Sensitizes Androgen-Dependent and -Independent Prostate Cancer to Radiotherapy via FASN/NF-κB Pathway
Source: Sci Rep. 2019 Sep 16;9:13284. doi: 10.1038/s41598-019-49486-2 (PMC6746859; doi:10.1038/s41598-019-49486-2)
Supplement: Supplementary file 1 — Supplemental information [file 41598_2019_49486_MOESM1_ESM.docx]

**Fatty Acid Inhibition Sensitizes Androgen-Dependent and -Independent Prostate Cancer to Radiotherapy via FASN/NF-κB Pathway**

Hui-Yen Chuang^a^, Yen-Po Lee^a^, Wei-Chan Lin^a, b, c^, Yi-Hsien Lin^d,e,*^, Jeng-Jong Hwang^a,f*^

^a^ Department of Biomedical Imaging and Radiological Sciences, National Yang-Ming University, Taipei, Taiwan 112

^b^ Department of Radiology, Cathay General Hospital, Taipei, Taiwan

^c^ School of Medicine, Fu-Jen Catholic University, New Taipei City, Taiwan

^d^ Division of Radiotherapy, Cheng Hsin General Hospital, Taipei, Taiwan

^e^ School of Medicine, National Yang-Ming University, Taipei, Taiwan;

^f^Department of Medical Imaging, Chung Shan Medical University Hospital, Taichung, Taiwan

***Corresponding authors:**

Jeng-Jong Hwang, PhD, Professor, Department of Biomedical Imaging and Radiological Sciences, National Yang-Ming University

No. 155, Sec. 2, Li-Nong St, Beitou, Taipei 112, Taiwan

Tel: +886-2-28267064; Fax: +886-2-28201095; Email: jjhwang@ym.edu.tw

Yi-Hsien Lin, MD, PhD, Division of Radiotherapy, Cheng Hsin General Hospital

No. 45, Cheng Hsin St, Beitou, Taipei 112, Taiwan

Tel: +886-2-28264400 ext 5750; Fax: +886-2-28264524; Email: ch9145@chgh.org.tw

**Supplemental figures**

**Figure S1**

**
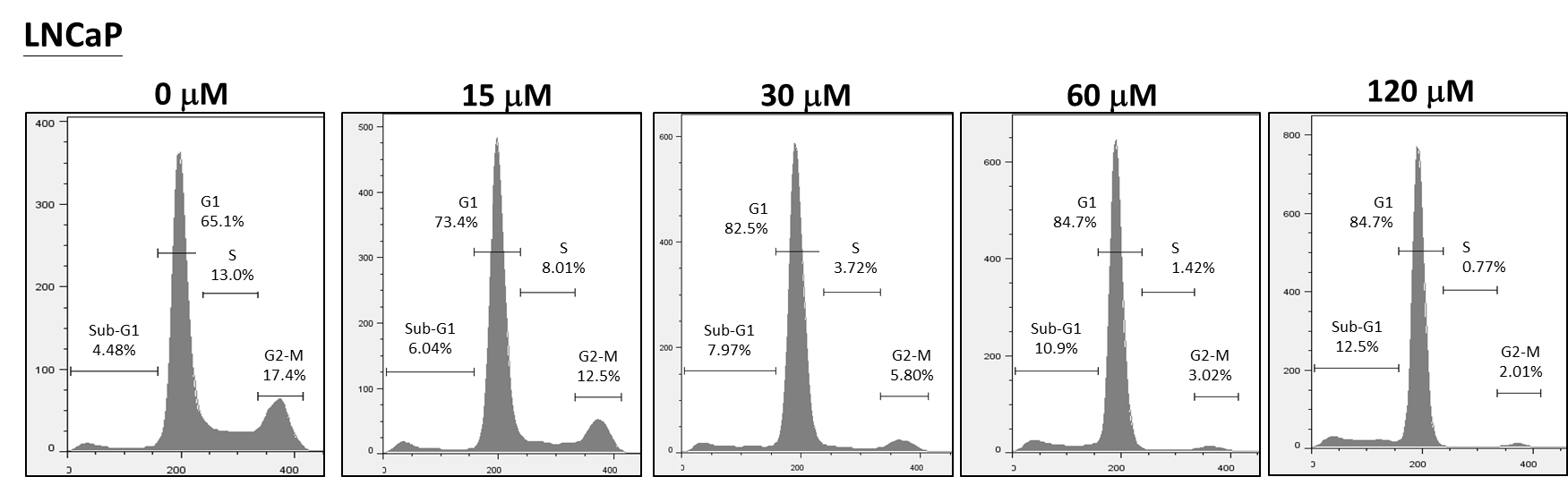
**


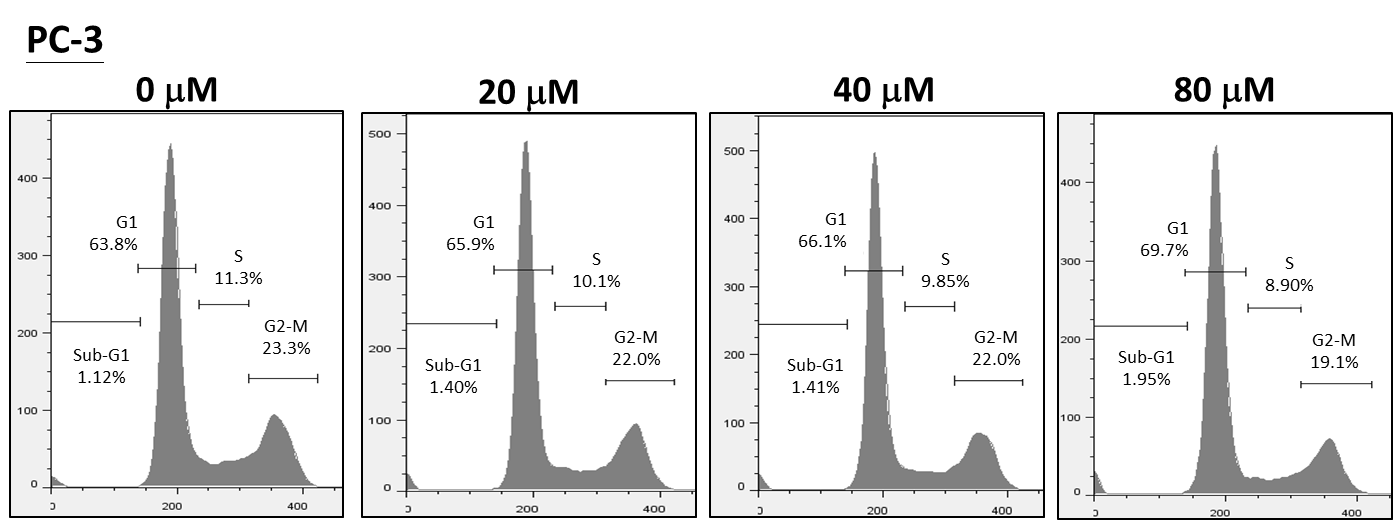


Figure S1. Representative cell cycle histograms obtained from cell cycle analysis using flow cytometry. (Upper panel) Orlistat resulted in significant G1 arrest, sub-G1 increases, and S and G2/M reductions in a dose-dependent manner in LNCaP cells. (Lower panel) Orlistat also caused G1 arrest and S and G2/M phase reductions in a dose-dependent manner in PC3 cell but did not raise the sub-G1 population as observed in LNCaP cells.

**Figure S2**


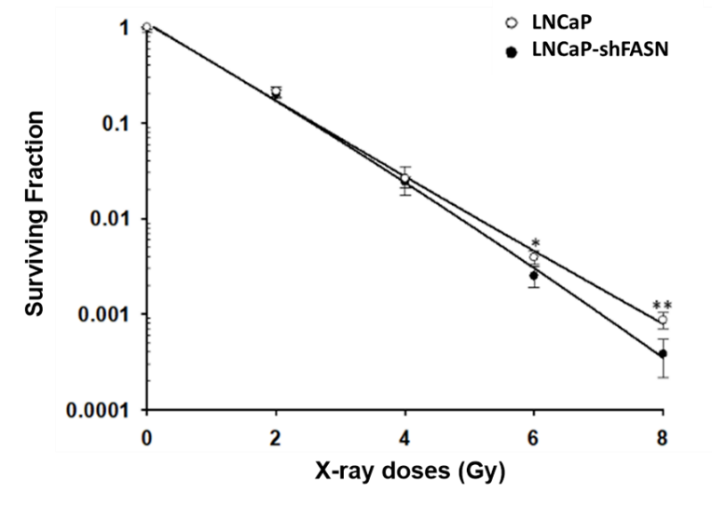


Figure S2. FASN expression decreased radiosensitivity in LNCaP cells. The radiosensitivity of both parental LNCaP and LNCaP-shFASN cells were determined by the colony formation assay. As shown in the figure, FASN inhibition increased the radiosensitivity of LNCaP cells when the cells were exposed to X-ray doses above 6 Gy. (*p < 0.05, **p < 0.01 as compared with the parental LNCaP cells)

**Figure S3**

**
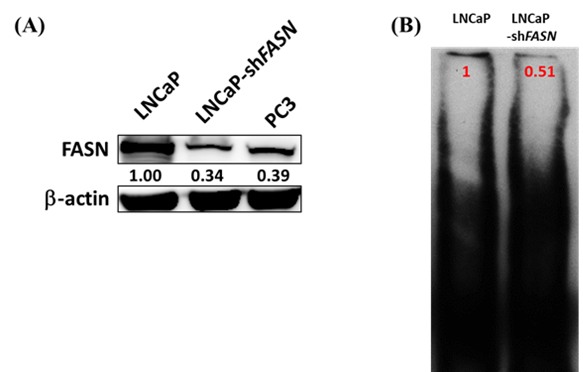
**

Figure S3. Comparisons of FASN expression levels in LNCaP, LNCaP-shFASN, and PC3 cells, and the NF-κB activity between LNCaP and LNCaP-shFASN cells. (A) FASN expressions in these three cell lines were determined by Western blotting. As shown in the figure, the parental LNCaP cells showed the most FASN expression, which was significantly decreased in the LNCaP-shFASN. Besides, PC3 cells expressed less FASN protein compared with parental LNCaP cells. (B) Knockdown of FASN repressed NF-κB activity in LNCaP cells.

**Figure S4**


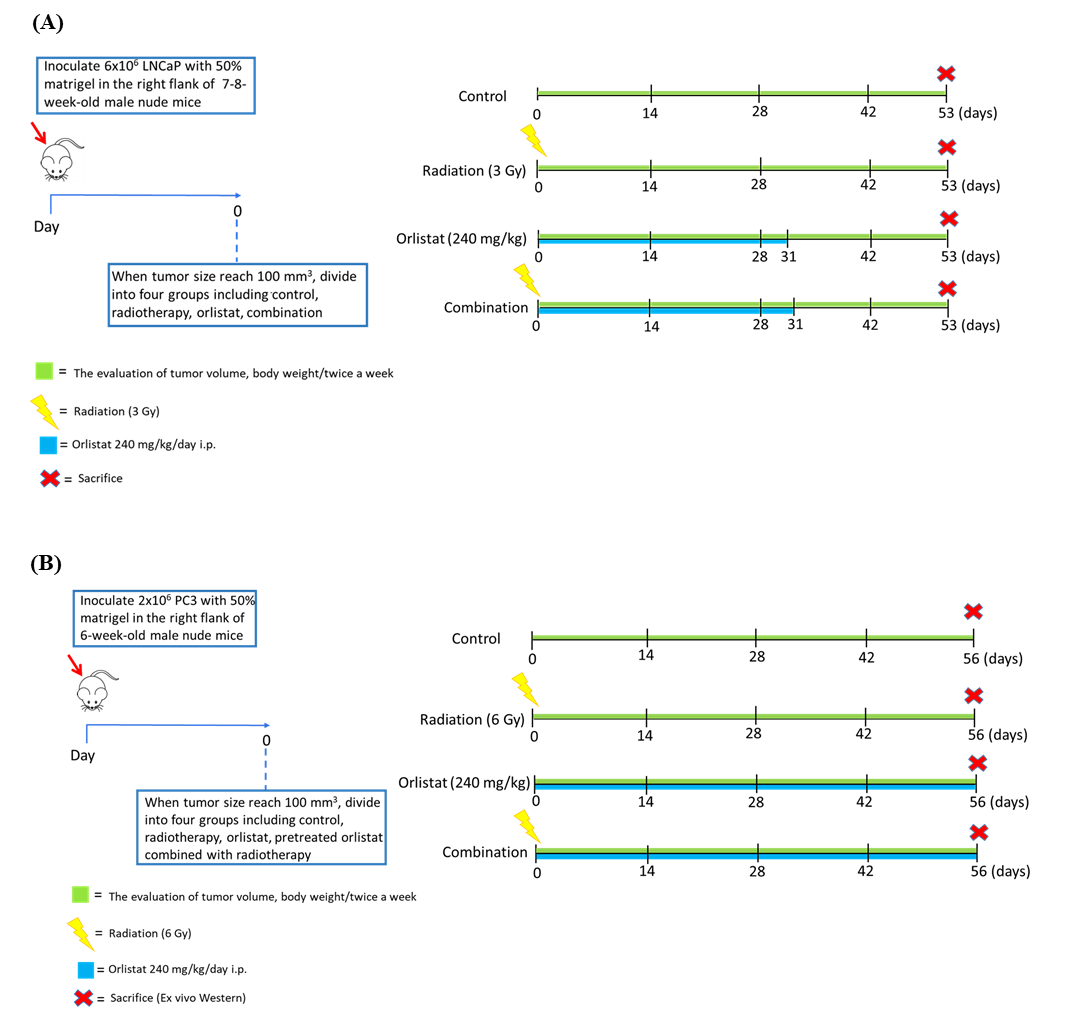


Figure S4. *In vivo* experimental illustrations. 6$\times$10^6^ LNCaP or 2$\times$10^6^ PC3 cells mixed with Matrigel were implanted into the right thigh of the 6-8-week-old male mice. Mice received different treatments one day after randomly divided into four groups. The mice in the ORL and the CTRL group received intraperitoneally (*i.p.*) 240 mg/kg/day orlistat and the same volumes of solvent every day, respectively. The RT group was treated with a single dose of 3 Gy for LNCaP and 6 Gy for PC3 tumours one day after divided into four groups. The COMB group of LNCaP and PC3 tumor-bearing mouse models received the first dose of orlistat 2 hours and 3 days before the irradiation, respectively. Tumour sizes and body weight were tracked twice a week.

**Figure S5**


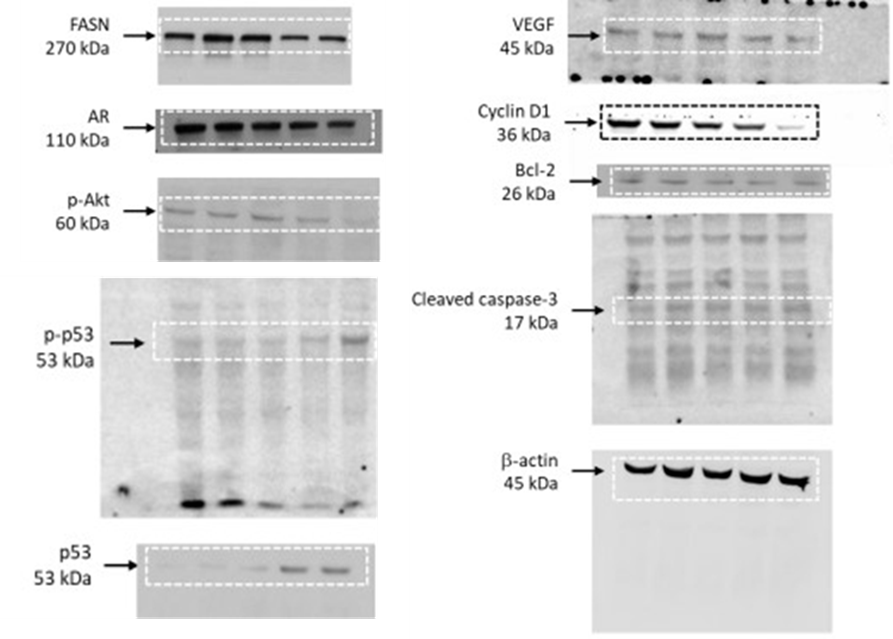


Figure S5: Originally blot images presented in Figure 3A

**Figure S6**


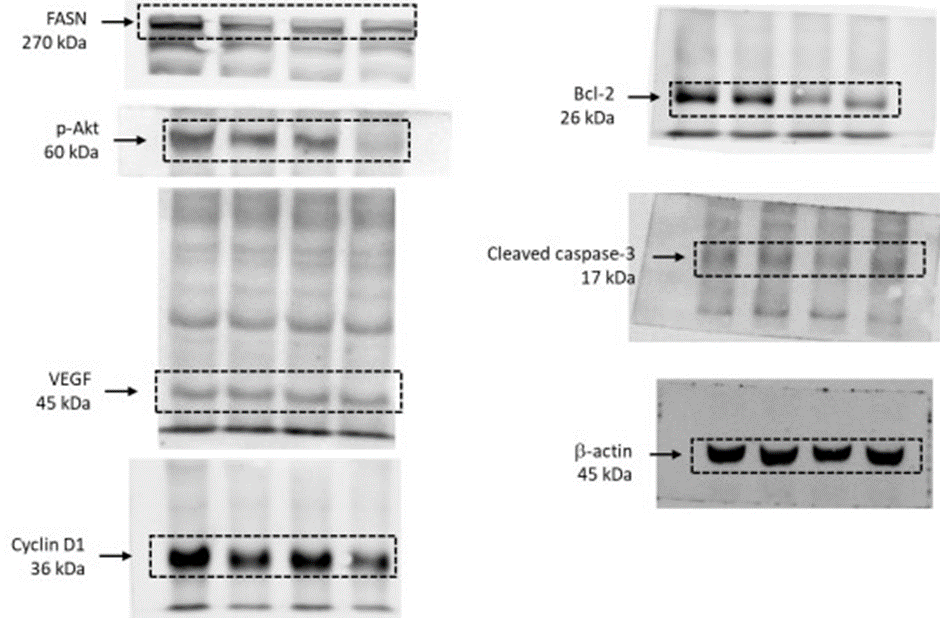


Figure S6: Originally blot images presented in Figure 3B

**Figure S7**

**
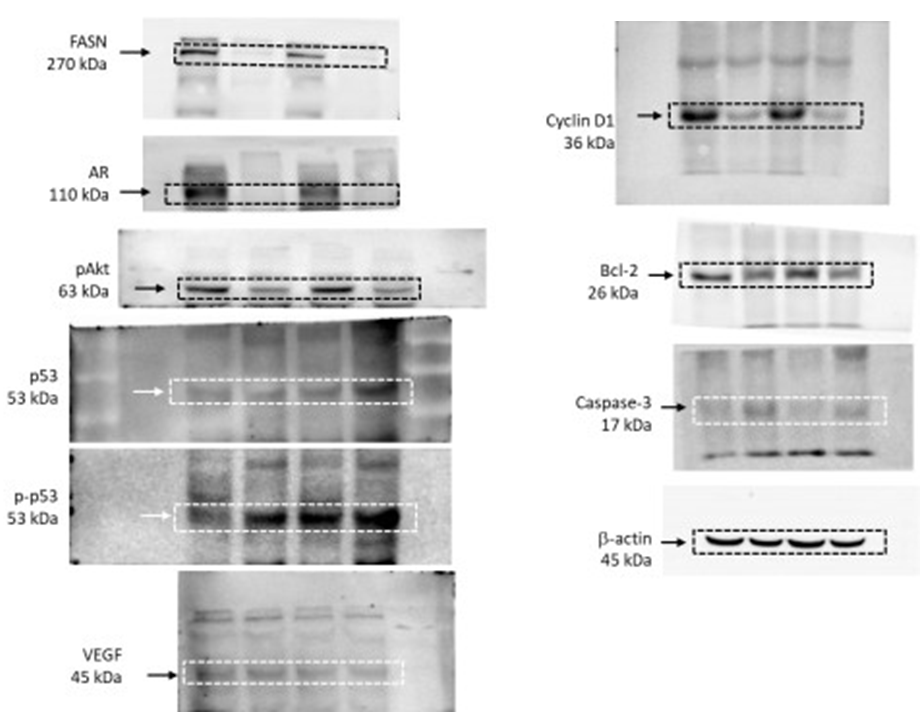
**

Figure S7: Originally blot images presented in Figure 4C

**Figure S8**

**
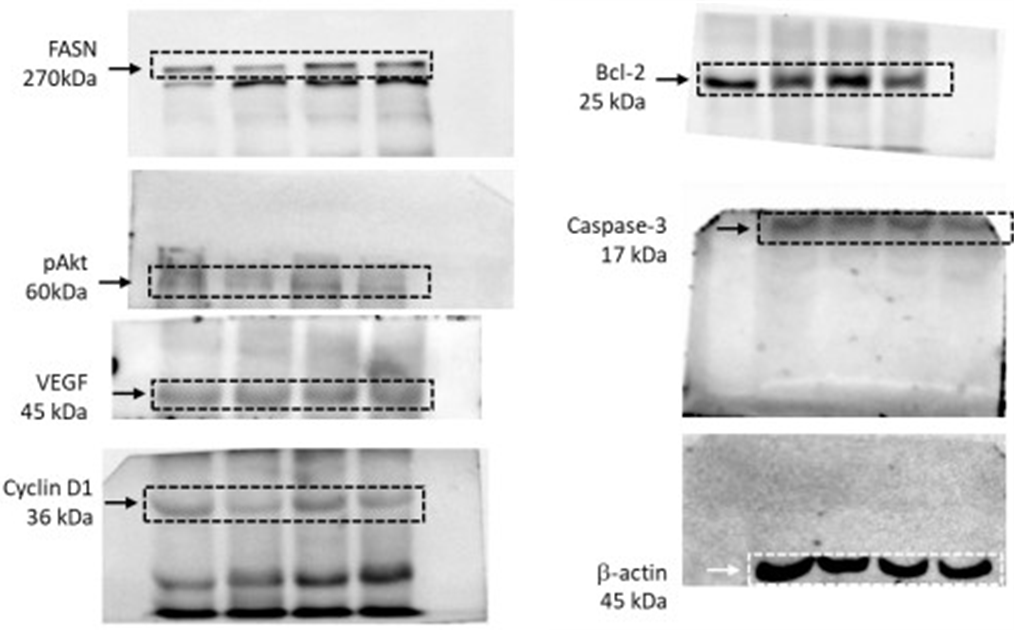
**

Figure S8: Originally blot images presented in Figure 4D
